# Identification MNS1, FRZB, OGN, LUM, SERP1NA3 and FCN3 as the potential immune-related key genes involved in ischaemic cardiomyopathy by random forest and nomogram

**DOI:** 10.18632/aging.204547

**Published:** 2023-02-27

**Authors:** Peng-Fei Zheng, Fen Liu, Zhao-Fen Zheng, Hong-Wei Pan, Zheng-Yu Liu

**Affiliations:** 1 Cardiology Department, Hunan Provincial People’s Hospital, Furong, Changsha 410000, Hunan, China; 2 Clinical Research Center for Heart Failure in Hunan Province, Furong, Changsha 410000, Hunan, China; 3 Institute of Cardiovascular Epidemiology, Hunan Provincial People’s Hospital, Furong, Changsha 410000, Hunan, China; 4 The First Affiliated Hospital of Hunan Normal University (Hunan Provincial People’s Hospital), Furong, Changsha 410000, Hunan, China

**Keywords:** ischaemic cardiomyopathy, random forest, immune cell infiltrates, bioinformatics analysis, immune-related genes

## Abstract

The immune molecular mechanisms involved in ischaemic cardiomyopathy (ICM) have not been fully elucidated. The current study aimed to elucidate the immune cell infiltration pattern of the ICM and identify key immune-related genes that participate in the pathologic process of the ICM. The differentially expressed genes (DEGs) were identified from two datasets (GSE42955 combined with GSE57338) and the top 8 key DEGs related to ICM were screened using random forest and used to construct the nomogram model. Moreover, the “CIBERSORT” software package was used to determine the proportion of infiltrating immune cells in the ICM. A total of 39 DEGs (18 upregulated and 21 downregulated) were identified in the current study. Four upregulated DEGs, including *MNS1*, *FRZB*, *OGN*, and *LUM,* and four downregulated DEGs, *SERP1NA3, RNASE2*, *FCN3* and *SLCO4A1,* were identified by the random forest model. The nomogram constructed based on the above 8 key genes suggested a diagnostic value of up to 99% to distinguish the ICM from healthy participants. Meanwhile, most of the key DEGs presented prominent interactions with immune cell infiltrates. The RT-qPCR results suggested that the expression levels of *MNS1*, *FRZB*, *OGN*, *LUM*, *SERP1NA3* and *FCN3* between the ICM and control groups were consistent with the bioinformatic analysis results. These results suggested that immune cell infiltration plays a critical role in the occurrence and progression of ICM. Several key immune-related genes, including the *MNS1*, *FRZB*, *OGN*, *LUM*, *SERP1NA3* and *FCN3* genes, are expected to be reliable serum markers for the diagnosis of ICM and potential molecular targets for ICM immunotherapy.

## INTRODUCTION

Ischaemic cardiomyopathy (ICM) refers to a disease in which the heart cannot pump blood normally due to long-term ischaemia and hypoxia damage to myocardial cells caused by severe coronary artery stenosis [1]. According to the World Health Organization, ICM is one of the leading causes of death worldwide. Moreover, heart failure (HF) secondary to ICM, in contrast to nonischaemic aetiologies, has been demonstrated to be independently associated with mortality [2, 3]. Due to the high hospitalization and mortality rates caused by coronary artery disease (CAD) and HF, prevention and timely therapy for ICM are especially crucial [4]. Presently, standardized drug therapy aimed at relieving symptoms and improving the prognosis is still the cornerstone of ICM treatment [5, 6]. However, even after complete revascularization for severe coronary artery stenosis and long-term standardized use of secondary prevention drugs for CAD, there are still some patients with a poor prognosis and deteriorating cardiac function, and immunologic and inflammatory responses may be partly responsible for the poor prognosis of these patients. It was reported that the pathophysiology of ICM involves a spectrum of metabolic, neurohumoural and inflammatory changes [6]. When damaged and necrotic cardiomyocytes die due to ischaemic shock to the myocardium, immune and nonimmune cells are activated [7]. Recently, Bansal et al. suggested that proinflammatory and antiangiogenic regulatory T-lymphocytes (Tregs) promote immune activation and pathological left ventricular remodelling in ischaemic heart failure [8]. Moreover, a previous study demonstrated that monocytes play a pathological role in mediating left ventricular remodelling, interstitial fibrosis, and progressive cardiac insufficiency [9]. Nehra et al. showed that CD8+ T cells were increased at eight weeks after myocardial infarction during ICM [10]. These evidences indicate that immune cells play a crucial role in the pathological process of ICM. Nevertheless, the pattern of immune cell infiltration in the ICM has not been fully elucidated. Therefore, further elucidating the pattern of immune cell infiltration in the ICM and identifying the key immune-related DEGs involved in the ICM will hopefully provide a new molecular target for ICM immunotherapy.

In recent years, with the rapid development of gene chip sequencing technology, microarray analysis has become a novel and practical method to screen susceptible genes involved in cardiovascular diseases [11, 12], which lays a solid foundation for establishing a new gene-based diagnostic model related to cardiovascular diseases. Random forest is a classification algorithm that uses a set of selected classification trees to perform gene selection and microarray data classification [13], which shows excellent performance even when most predictive variables are disordered [14, 15]. Importantly, the prediction error could be reduced by using the random forest algorithm on a selected subset of genes compared with the existing methods and other proposed methods [13, 16]. Moreover, compared with other methods, the random forest method is more effective in identifying key genes significantly associated with disease. [17]. Recently, as a widely applied analytical method, CIBERSORT has often been used to study the immune cell infiltration pattern in diseases based on RNA sequencing or microarray data and to evaluate the infiltrated proportion of 22 immune cells in each sample [18]. However, studies using a combination of random forest and CIBERSORT to verify immune-related genes associated with ICM are very limited. Hence, in the current study, CIBERSORT was used to evaluate the immune cell infiltration patterns in ICM, and the key DEGs were identified using random forest and then we further analysed the correlation between the key DEGs and immune cells. Finally, we verified the expression levels of the screened key DEGs and their diagnostic efficiency in the testing sets and the collected clinical samples.

## RESULTS

### Identification of DEGs

**Figure 1:**
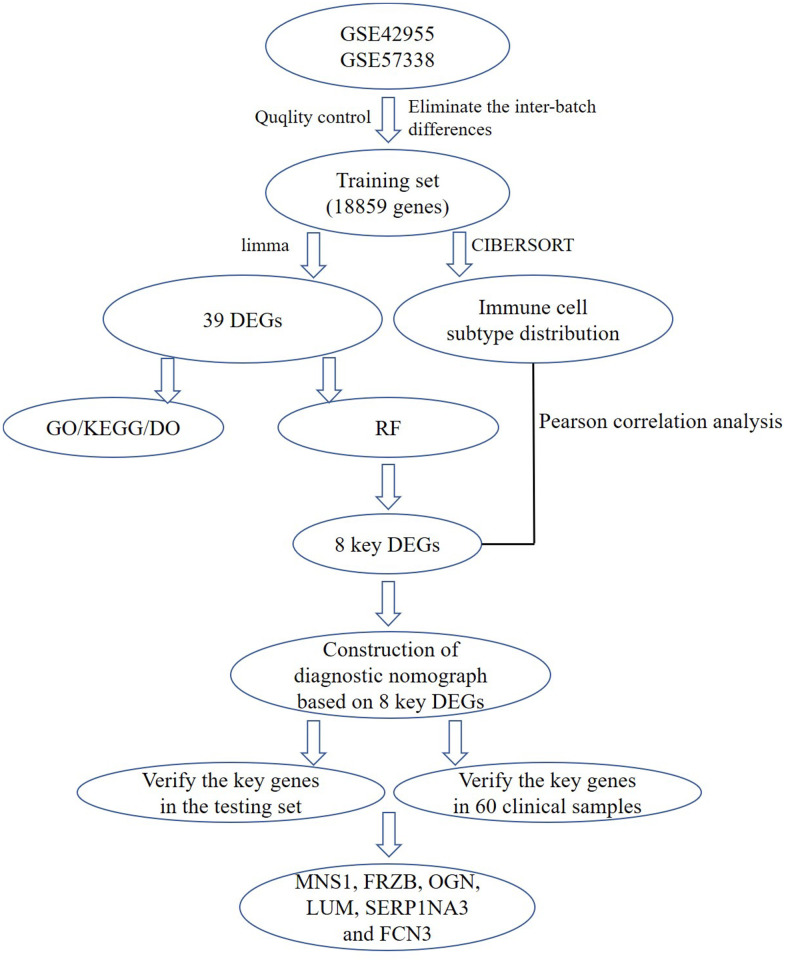
**A flow chart for analysis.** DEGs, differentially expressed genes; GO, gene ontology annotation; KEGG, kyoto encyclopedia of genes and genomes pathway enrichment analyses; DO, disease ontology analysis; RF, random forest; MNS1, meiosis-specific nuclear structural 1; FRZB, frizzled-related protein; OGN, osteoglycin; LUM: lumican; SERPINA3: serpin family A member 3; FCN3: ficolin-3.

**Figure 2:**
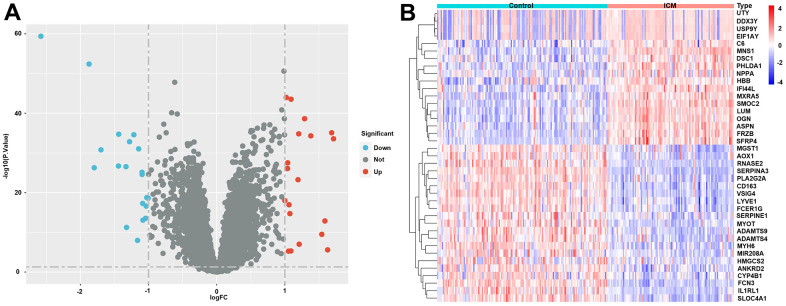
**Differentially expressed genes (DEGs) in ICM and healthy samples.** (**A**) Volcano plot of the 39 DEGs. Red dots represent significantly upregulated genes, and blue dots represent significantly downregulated genes. (**B**) Heatmap of the 39 lipid-related DEGs in ICM and control samples. Red blocks indicate high-expression genes, and blue blocks indicate low-expression genes.

The specific workflow is shown in Figure 1. After data normalization and removal of the batch differences, a total of 39 DEGs, including 18 upregulated and 21 downregulated DEGs, were identified (Supplementary Table 1) and could be visualized in the volcano plot and heatmap (Figure 2A, 2B). In addition, the specific expressions of 39 DEGs in the training set are also shown in the Supplementary Table 2.

### Functional analysis

**Figure 3:**
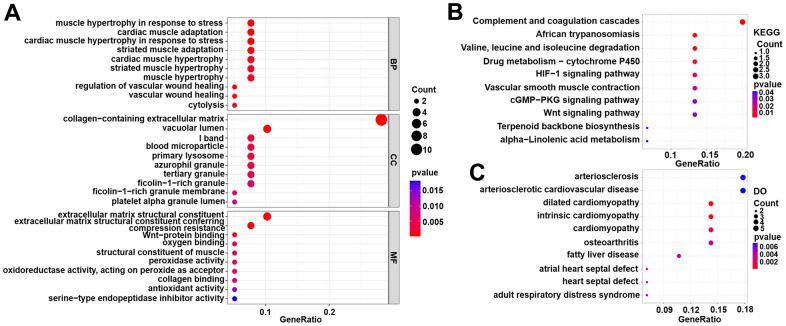
**Functional enrichment analyses for DEG genes in the training set.** (**A**) Gene Ontology (GO) enrichment analysis. (**B**) Kyoto Encyclopedia of Genes and Genomes (KEGG) pathway analysis. (**C**) Disease Ontology (DO) analysis.

As shown in Figure 3A, the results of the GO analysis include biological processes, cellular components and molecular function. The top three enriched GO terms were muscle hypertrophy in response to stress, cardiac muscle adaptation, and cardiac muscle hypertrophy in response to stress (biological processes); collagen-containing extracellular matrix, vacuolar lumen, and I band (cellular components); extracellular matrix structural constituent, extracellular matrix structural constituent conferring, and compression resistance (molecular function). The KEGG enrichment analyses suggested that DEGs were involved in the following pathways, including the complement and coagulation cascades, the HIF-1 signalling pathway, the cGMP-PKG signalling pathway, and the Wnt signalling pathway (Figure 3B). The DO analysis also suggested that the DEGs were mainly concentrated in some cardiovascular system diseases such as cardiomyopathy, arteriosclerosis, arteriosclerosis cardiovascular disease, intrinsic cardiomyopathy, heart septal defect, and dilated cardiomyopathy (Figure 3C). In addition, the detailed results of the KEGG, DO and GO enrichment analyses are presented in the Supplementary Tables 4, 5.

### Screening the key DEGs with the random forest classification model

**Figure 4:**
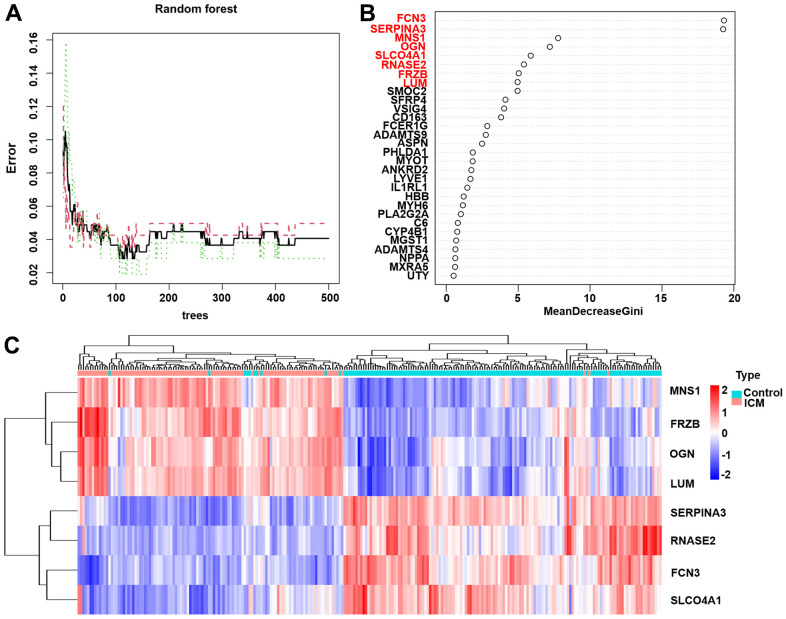
**The results of the top 8 DEGs screened by random forest.** (**A**) The plot of performance in log scale against epoch number. (**B**) The importance of the top 8 DEGs ranked by the mean decrease in accuracy. (**C**) Heatmap of the top 8 genes. Red blocks indicate high-expression genes, and blue blocks indicate low-expression genes.

The out-of-bag classification error was relatively stable when the decision tree was set at 450 in the random forest classification model (Figure 4A). A total of 15 DEGs were identified as potential diagnostic markers of ICM using the random forest algorithm (Mean Decrease Gini >2) and the top 8 DEGs (*MNS1*, *FRZB*, *OGN*, *LUM*, *SERP1NA3*, *RNASE2*, *FCN3*, and *SLCO4A1*) were selected as key genes for further analysis (Figure 4B). In addition, the specific importance of 39 DEGs in the training set are also shown in the Supplementary Table 3. As shown in Figure 4C, the 8 key DEGs might be useful to distinguish between the ICM and control samples. Among them, *MNS1*, *FRZB*, *OGN*, and *LUM* were upregulated while *SERPINA3*, *RNASE2*, *FCN3*, and *SLCO4A1* were downregulated in the control samples compared with the ICM samples.

### ICM nomogram construction and verification

**Figure 5:**
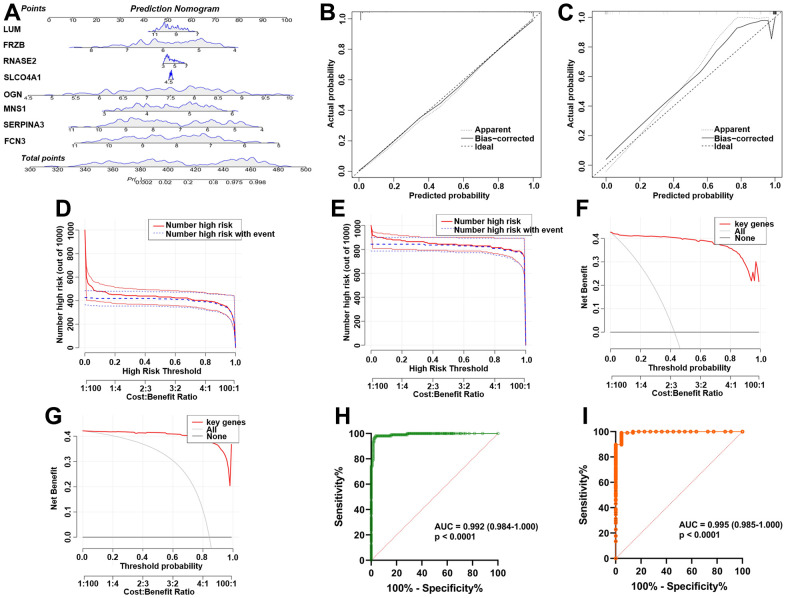
**Establishment and verification of the ICM diagnostic signature and nomogram.** (**A**) Eight predictive nomograms of genetic diagnosis. (**B**) Calibration curve in the training set. (**C**) Calibration curve in the testing set. (**D**) Decision curve analysis (DCA) in the training. (**E**) DCA in the testing set. (**F**) Clinical impact curve in the training set. (**G**) Clinical impact curve in the testing set. (**H**) ROC curve in the training set. (**I**) ROC curve in the testing set.

The nomogram (Figure 5A) was constructed from the results of the multiple logistic regression to intuitively understand the relationship between 8 key genes and the prognosis of ICM. The calibration curves were plotted based on the expression levels of the 8 key genes in the training (Figure 5B) and validation (Figure 5C) sets, respectively, to verify the accuracy of the nomogram prediction. The predicted curves were almost the ideal curve, which suggested good performance. The DCA confirmed the improved clinical utility of the nomogram in predicting the morbidity of ICM patients in the training (Figure 5D) and validation (Figure 5E) sets. Moreover, the clinical impact curves also demonstrated that the nomogram model had significant predictive ability and good clinical utility both in the training (Figure 5F) and validation (Figure 5G) sets. In addition, the area under the curve (AUC) was 0.992 (95% confidence interval (CI) 0.984-1.000) in the training (Figure 5H) and 0.995 (95% CI 0.985-1.000) in the validation (Figure 5I) sets.

### Immune cell distribution pattern

**Figure 6:**
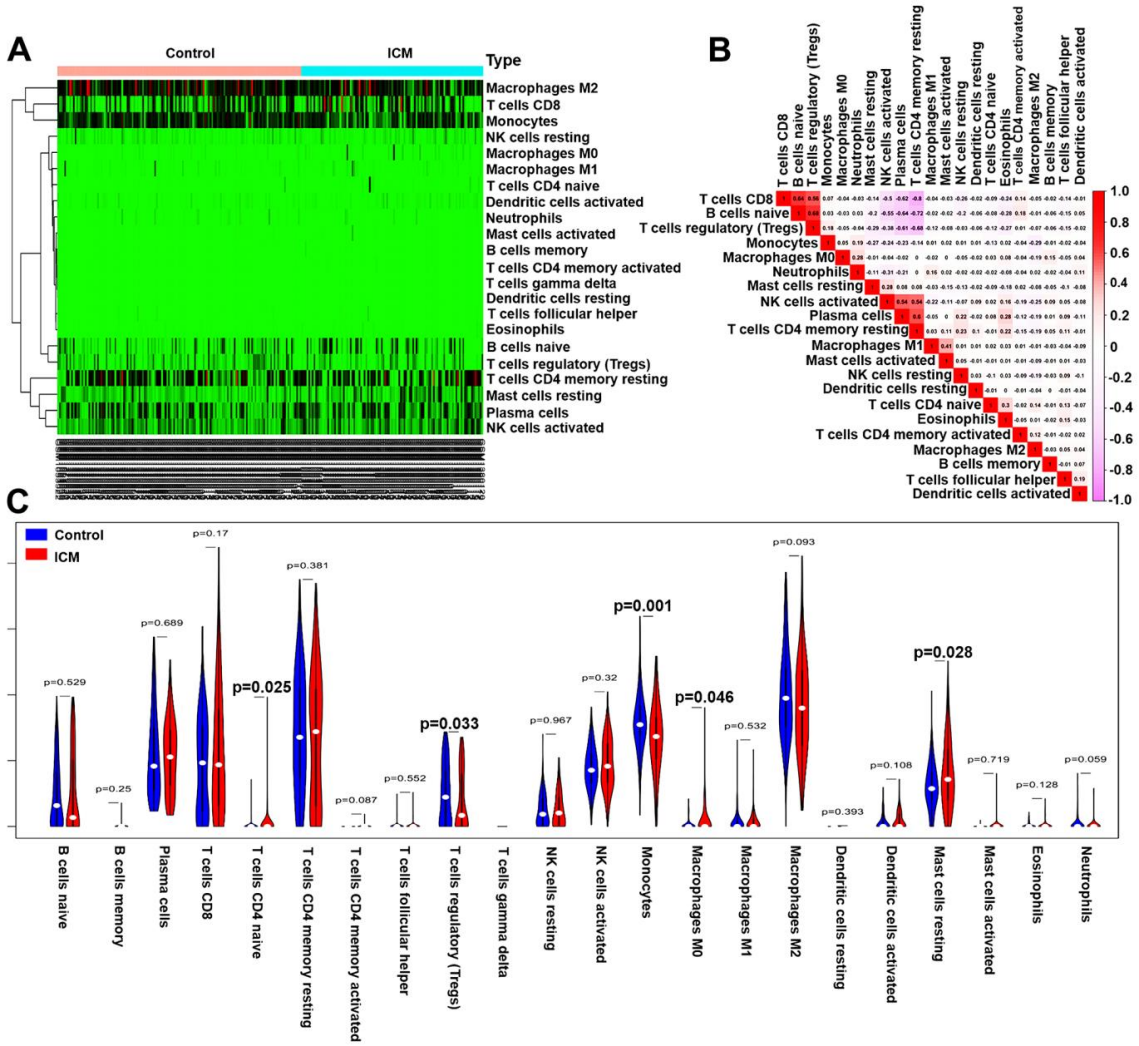
**Pattern of immune cell subtype infiltration in the training set.** (**A**) Heatmap of the 22 immune cell proportions in each sample. (**B**) Correlation heatmap of the 22 immune cells. The deeper the red, the stronger the positive correlation. The darker the pink, the stronger the negative correlation. (**C**) Representative violin plot showing the different fractions of infiltrated immune cells.

The immune fractions expressed differentially between the control and ICM samples were evaluated using the CIBERSORT algorithm. The histogram intuitively shows that the total proportion of 22 different immune cell subtypes in 246 ICM samples was 1 (Supplementary Figure 3). The heatmap (Figure 6A) suggested that the proportions of several immune cells in both the ICM and control samples were obviously different. Based on the correlation matrix, we found that CD4 memory resting T cells were negatively correlated with Tregs and CD8 T cells but positively correlated with plasma cells; Tregs were positively related to CD8 T cells and naive B cells but negatively related to plasma cells (Figure 6B). As shown in Figure 6C, naive CD4 T cells, M0 macrophages and resting mast cells were increased, while Tregs and monocytes were decreased in ICM samples compared with control samples (*p* <0.05). Furthermore, Supplementary Table 6 shows the details of the immune cell infiltration pattern between the ICM and control samples.

**Figure 7:**
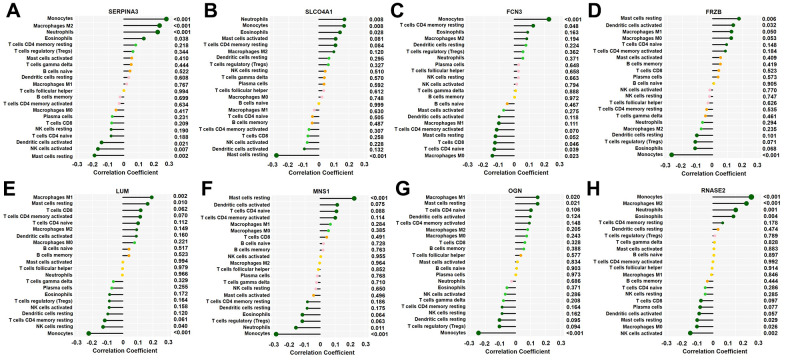
**Correlation between 8 key genes and infiltrated immune cells.** Representative graphs showing the correlation between the infiltrated immune cells and *SERP1NA3* (**A**), *SLCO4A1* (**B**), *FCN3* (**C**), *FRZB* (**D**), *LUM* (**E**), *MNS1* (**F**), *OGN* (**G**) and *RNASE2* (**H**). The correlation strength is shown by the dot size; the *p* values are expressed as the changes in dot colour, and *p* < 0.05 was considered statistically significant. SERPINA3: serpin family A member 3; SLCO4A1, solute carrier organic anion transporter family member 4A1; RNASE2, ribonuclease A family member 2; FCN3, ficolin-3; FRZB, frizzled-related protein; LUM, lumican; MNS1, meiosis-specific nuclear structural 1; OGN, osteoglycin.

The correlations between the 8 key DEGs and infiltrated immune cells are shown in Figure 7. The *SERPINA3* gene was negatively correlated with resting mast cells, activated dendritic cells, and activated NK cells but positively correlated with monocytes, M2 macrophages, eosinophils, and neutrophils; the *SLCO4A1* gene was negatively correlated with resting mast cells and positively correlated with neutrophils, monocytes, and eosinophils; the *FCN3* gene was positively correlated with monocytes and resting memory CD4 T cells but negatively correlated with M0 macrophages, CD8 T cells and naive CD4 T cells; the *FRZB* was positively correlated with resting mast cells and activated dendritic cells but negatively correlated with monocytes; the *LUM* gene was positively correlated with M1 macrophages and resting mast cells but negatively correlated with monocytes and resting NK cells; the *MNS1* gene was positively correlated with resting mast cells but negatively correlated with neutrophils and monocytes; the *OGN* gene was positively correlated with M1 macrophages and resting mast cells but negatively correlated with monocytes; and the *RNASE2* gene was positively correlated with monocytes, neutrophils, esoinophils and M2 macrophages but negatively correlated with activated NK cells, M0 macrophages, and resting mast cells (*p*<0.05–0.01, respectively).

### External validation of the top 8 key DEGs in the testing set

The expression levels of these 8 key DEGs are shown in Supplementary Figure 4. The expression levels of *MNS1* (*p* = 0.00033), *FRZB* (*p* = 6.3e-09), *OGN* (*p* = 2.9e-08), and *LUM* (*p* = 2.3e-10) were increased in ICM compared to the control samples, while *SERP1NA3* (*p* = 7.2e-09), *RNASE2* (*p* = 0.0043), *FCN3* (*p* = 2.9e-12), and *SLCO4A1* (*p* = 7.2e-09) were decreased in the ICM samples.

### Validation by RT-qPCR

**Figure 8:**
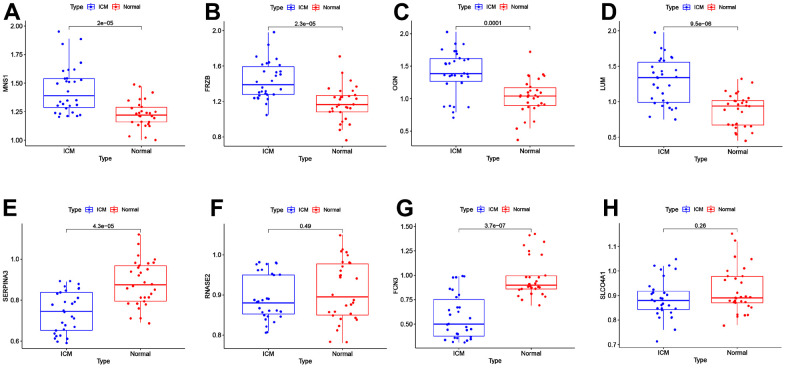
**External validation of the key genes in the clinical samples.** The expression levels of *MNS1* (**A**), *FRZB* (**B**), *OGN* (**C**), *LUM* (**D**), *SERPINA3* (**E**), *RNASE2* (**F**), *FCN3* (**G**) and *SLCO4A1* (**H**) in the clinical samples. MNS1, meiosis-specific nuclear structural 1; FRZB, frizzled-related protein; OGN, osteoglycin; LUM: lumican; SERPINA3: serpin family A member 3; FCN3: ficolin-3; SLCO4A1, solute carrier organic anion transporter family member 4A1; RNASE2, ribonuclease A family member 2.

**Figure 9:**
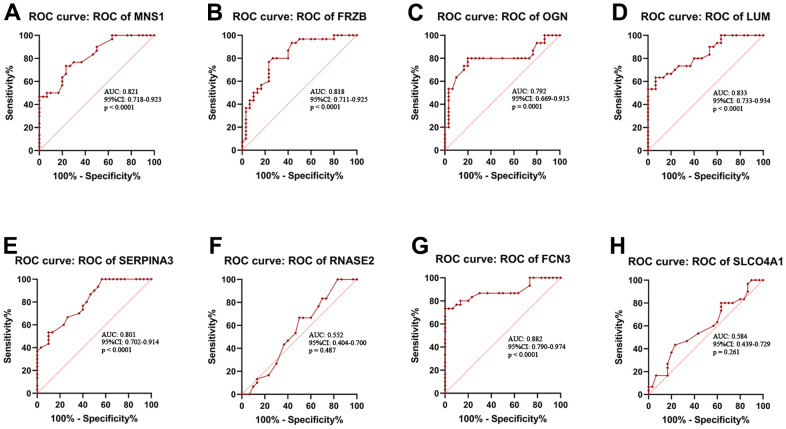
**Receiver operating characteristic (ROC) curve analysis.** ROC curve analysis of *MNS1* (**A**), *FRZB* (**B**), *OGN* (**C**), *LUM* (**D**), *SERPINA3* (**E**), *RNASE2* (**F**), *FCN3* (**G**) and *SLCO4A1* (**H**) in the clinical samples. MNS1, meiosis-specific nuclear structural 1; FRZB, frizzled-related protein; OGN, osteoglycin; LUM: lumican; SERPINA3: serpin family A member 3; FCN3: ficolin-3; SLCO4A1, solute carrier organic anion transporter family member 4A1; RNASE2, ribonuclease A family member 2.

The expression levels of *MNS1* (*p* = 2e-05), *FRZB* (*p* = 2.3e-05), *OGN* (*p* = 0.0001), and *LUM* (*p* = 9.5e-06) were increased in ICM compared to the control group, and *SERPNA3* (*p* = 4.3e-05) and *FCN3* (*p* = 3.7e-07) were higher in the control group (Figure 8). Nevertheless, the expression levels of *RNASE2* (*p* = 0.49) and *SLCO4A1* (*p* = 0.23) were similar between the two groups. As shown in Figure 9, the AUC values of *MNS1*, *FRZB*, *OGN*, *LUM*, *SERP1NA3*, and *FCN3* were 0.821 (95% CI: 0.718–0.923), 0.818 (95% CI: 0.711–0.925), 0.792 (95% CI: 0.669–0.915), 0.833 (95% CI: 0.733–0.934), 0.808 (95% CI: 0.701–0.914), and 0.882 (95% CI: 0.790–0.974), respectively.

## DISCUSSION

Ischaemic cardiomyopathy (ICM) is a chronic immune system activation state, and immune or inflammatory mechanisms play crucial roles in its occurrence and development [8]. After ischaemic injury of myocardial tissue, the inflammatory immune response will activate the repair process, which is manifested as the removal of necrotic tissue debris, the formation of stable scars, and the initiation of neovascularization in the myocardium to achieve effective wound healing. However, incomplete immune resolution or a persistent low-grade inflammatory response can lead to poor remodelling of myocardial tissue and left ventricular dilatation, ultimately leading to ICM [10]. Scientists have observed that the innate inflammatory cascade is primarily mediated by neutrophils, monocytes, and macrophages, and it contributes to chronic inflammatory processes [19]. Notably, monocytes and macrophages have been found to play complex and diverse roles in myocardial injury [10]. He et al. showed that monocytes and reparative macrophages contribute to the removal of necrotic debris, granulation tissue formation, and angiogenesis in the early stage after myocardial infarction [20]. However, Ismahil et al. demonstrated that monocytes play a pathological role in mediating left ventricle remodelling, interstitial fibrosis and progressive cardiac dysfunction [9]. Depending on the stimulation, M0 macrophages polarize into M1 and M2 macrophages, which perform proinflammatory and anti-inflammatory functions, respectively [21]. In addition, other types of immune cells have also been found to play key roles in ventricular remodelling or heart failure. Sun et al. demonstrated that TNF-α secreted by mast cells could induce matrix metalloproteinase activation, leading to ventricular remodelling [22]. In heart failure, chymotrypsin released by mast cells causes left ventricle dysfunction by promoting cardiomyocyte apoptosis and fibroblast proliferation [23]. CD4+ T cells impart proinflammatory and detrimental effects during chronic ischaemic heart failure and promote adverse ventricular remodelling [24, 25]. Tregs are the most critical immune cells found *in vivo* with powerful immune-suppressive potential [10]. A persuasive study indicated that Tregs are essential for modulating immune responses, promoting cardiac repair and initiating immune resolution [26]. It has been reported that adoptive transfer of Tregs after myocardial infarction can alleviate inflammation and promote cardiac repair. Importantly, partial depletion and successful reconstruction of Tregs resets their phenotypic and immunosuppressive potential, improving cardiac function, reducing left ventricle remodelling and partially reversing the ICM [27]. These studies indicate that the immune microenvironment plays a complex and critical role in the occurrence and development of ICM. Nevertheless, the immune microenvironment of ICM has not been fully elucidated.

In the current research, we found that Treg and monocyte infiltration were decreased and that naive CD4 T cells, resting mast cells and M0 macrophages were increased in the ICM compared with normal samples, which showed that Tregs and monocytes might play protective roles and that naive CD4 T cells, resting mast cells and M0 macrophages might play promoting roles in the pathological process of ICM. Moreover, several interactions among 22 types of infiltrating immune cells in the ICM were also noted. We found that CD4 memory resting T cells were negatively correlated with Tregs and CD8 T cells but positively correlated with plasma cells; Tregs were positively correlated with CD8 T cells and naive B cells but negatively correlated with plasma cells. However, our results have only partially elucidated the characteristics of the immune microenvironment in ICM, and the immune-related key molecular targets involved in ICM have not been fully identified. Therefore, identifying these molecular markers will provide a new perspective for the diagnosis and immunotherapy of ICM. In this study, a total of 8 key immune-related DEGs (*MNS1*, *FRZB*, *OGN*, *LUM*, *SERP1NA3*, *RNASE2*, *FCN3*, and *SLCO4A1*) involved in ICM were screened by a random forest algorithm combined with a nomogram model. Meanwhile, a nomogram based on the above 8 key genes suggested a diagnostic value of up to 99% to distinguish ICM from healthy participants. In addition, the results of RT-qPCR and ROC curve analysis showed that the expression levels of *MNS1*, *FRZB*, *OGN*, and *LUM* were increased in ICM patients compared with the control group, while the expression levels of *SERP1NA3* and *FCN3* were decreased. Moreover, gene enrichment analysis showed that the potential molecular mechanism of these key genes involved in ICM was mainly related to inflammation or immunity, but the underlying molecular mechanisms of these genes might be slightly different.

Among the four upregulated genes in ICM, frizzled-related protein (*FRZB*) is a secreted protein that serves as a modulator of the Wnt signalling pathway by directly interacting with Wnt and it plays a key role in dorsoventral patterning of the mesoderm during vertebrate development [28]. It has been demonstrated that *FRZB* is a key molecule in the progression of abdominal aortic aneurysm [29], and it can reduce the growth and aggressiveness of fibrosarcoma cells [30]. Previous research has reported that *FRZB* is involved in congenital heart defects [31]. Yang et al. [32] suggested that *FRZB* could regulate dilated cardiomyopathy through the extracellular matrix signalling pathway. Meanwhile, Ma et al. [33] showed that *FRZB* was significantly upregulated in hypertrophic cardiomyopathy (HCM) and can serve as a biomarker and is a potential therapeutic target for HCM. This evidence strongly suggests that FRZB is significantly associated with cardiomyopathy development, but the relationship between *FRZB* and immune cells and ICM remains unclear. Our current study suggested that *FRZB* was upregulated in ICM samples; meanwhile, we also observed that was negatively related to monocytes but positively related to resting mast cells and activated dendritic cells. Nevertheless, further research is needed to confirm these discoveries.

Osteoglycin (*OGN*) is a protein that belongs to the small leucine-rich proteoglycan (SLRP) family, and these SLRPs play critical roles in shaping the organization and structure of the extracellular matrix in the heart and other organs [34]. In 2008, *OGN* was first discovered to be related to the ventricular mass [35], which laid a foundation for further research on the role of OGN in cardiovascular disease. A study showed that *OGN* expression was increased in patients with coronary heart disease compared with normal subjects and was related to the severity of the coronary lesions [36]. Rienks et al. demonstrated that 72 kDa chondroitin sulfate-OGN could aggravate cardiac inflammation by enhancing Toll-like receptor 4 activation [34]. It has been reported that the downregulation of *OGN* is beneficial for the formation of endothelial cells and it might become a novel treatment target for ischaemic vascular diseases [37]. In addition, *OGN* expression changes during the course of cytotoxicity events, and chemotactic stimulation was found in natural killer cells and neutrophils [38], but the relationship between *OGN* and immune cells and ICM susceptibility remains poorly understood. Herein, we noticed that the expression of *OGN* was significantly increased in ICM patients compared with healthy subjects and it was negatively related to monocytes but positively related to M1 macrophages and resting mast cells. Thus, we speculate that the upregulation of *OGN* expression might cause ICM by enhancing immune inflammation.

Lumican (*LUM*) belongs to the small leucine-rich repeat proteoglycan family [39, 40]. Previous studies reported that *LUM* could regulate the growth of cardiomyocytes by adjusting the pericellular extracellular matrix [41], and moderate *LUM* deficiency can weaken cardiac fibrosis and ameliorate diastolic dysfunction after excessive pressure load [42]. When heart failure occurs, fibroblasts produce large amounts of *LUM*, which is involved in cardiac remodelling processes induced by mechanical and proinflammatory stimulation [43]. Moreover, Zhang et al. [44] suggested that *LUM* may have a potentially good predictive ability for the diagnosis of dilated cardiomyopathy. The meiosis-specific nuclear structural 1 (*MNS1*) gene is involved in bile acid, fatty acid, and haem metabolism [45] and it affects the course of heart failure. A recent study showed that *MNS1* was screened through three machine learning methods to be regarded as a possible bioinformatics marker for heart failure [46]. These results indicate that *LUM* and *MNS1* play vital roles in heart failure, but their association with the immune cells involved in ICM is still unclear. In the current research, we found that *LUM* and *MNS1* were highly expressed in ICM patients compared with healthy subjects. Meanwhile, we also noticed that *LUM* and *MNS1* were positively correlated with resting mast cells but negatively correlated with monocytes. These findings suggested that *LUM* and *MNS1* may participate in ICM by affecting resting mast cells and monocyte cells, but more studies are needed to verify our current findings.

Serpin family A member 3 (*SERPINA3*), an acute phase response gene, is upregulated during the process of inflammation [47] and has been demonstrated to promote myocardial ischaemia reperfusion injury [48]. Wågsäter et al. suggested that *SERPINA3* expression is significantly increased in human atherosclerotic lesions [49]. However, a recent study found that *SERPINA3* expression was decreased in heart failure patients compared with control subjects, indicating that *SERPINA3* played a protective role during the process of heart failure [7]. Meanwhile, Masanori et al. showed that *SERPINA3* might serve as a potential prognostic biomarker for heart failure [50]. Ficolin-3 (*FCN3*) is a recognition molecule in the lectin pathway of the complement system that is expressed in the liver and the lung [51]. Trine et al. demonstrated that *FCN3* is a serum protein that may be involved in the progression of systemic lupus erythematosus [52]. Shang et al. found that *FCN3* may activate the complement system and be overexpressed in type 2 diabetes plasma [53]. However, Song et al. showed that *FCN3* expression was significantly decreased in ICM patients compared with healthy subjects [54]. These studies indicate that *SERPINA3* and *FCN3* maintain different expression patterns in different stages of atherosclerosis and related diseases. Similarly, in the current study, the expression levels of *SERPINA3* and *FCN3* were significantly decreased in ICM patients compared with healthy subjects. Moreover, *SERPINA3* is positively related to neutrophils and monocytes but negatively related to activated NK cells; *FCN3* is positively related to monocytes but negatively related to M0 macrophages, CD8 T cells and naive CD4 T cells. Our findings indicated that *SERPINA3* and *FCN3* might play a protective role in the pathological process of ischaemic cardiomyopathy by regulating immune cell infiltration.

However, our current study also has some limitations. First, the included clinical samples were relatively small; therefore, our conclusions must be verified by a larger ICM cohort. Second, we did not detect the level of immune-related cytokines or chemokines in clinical samples, so we could not further analyze the correlation between these key genes and cytokines and chemokines. Third, the key DEGs were only confirmed in clinical samples, and their potential roles were not demonstrated in ICM cells or animal models. Hence, more *in vivo* and *in vitro* studies are needed to clarify the association between the 6 key genes and the infiltrated immune cells and to understand the mechanism of these key genes during the pathological process of ICM.

In conclusion, we confirmed that *MNS1*, *FRZB*, *OGN*, *LUM*, *SERP1NA3*, and *FCN3* are novel credible serum markers for the diagnosis of ICM using random forest combined with nomogram. We noticed that naive CD4 T cells, M0 macrophages and resting mast cells may be correlated with the occurrence and progression of ICM, while Tregs and monocytes may play a protective role in ICM. The interaction mechanisms between the above key DEGs and immune cells may be of great significance for the pathogenesis and progression of ICM.

## MATERIALS AND METHODS

### Data download and preprocessing

The Gene Expression Omnibus (GEO) website was used to download 4 ICM-related datasets (https://www.ncbi.nlm.nih.gov/geo/). The GSE42955 dataset, including 12 ICM and 5 control samples, was based on the GLP6244 platform (Affymetrix Human Gene 1.0 ST Array). GSE57338 included 95 ICM and 136 control samples and was retrieved from GPL11532 (Affymetrix Human Gene 1.1 ST Array). Based on the GLP96 platform (Affymetrix Human Genome U 133A Array), we also downloaded the GSE1869 dataset (including 10 ICM and 5 control samples) and the GSE5406 dataset (including 108 ICM and 16 controls) for subsequent validation. As shown in Supplementary Figure 1, the *normalize Between Arrays* function in the “limma*”* package was used to normalize samples, and the interbatch differences were eliminated using the “sva” package. Moreover, after removing two outlier samples (GSM1380018 and GSM1379815) (Supplementary Figure 2), the key genes involved in ICM were identified using a total of 246 samples in the training sets (GSE42955 combined with GSE57338). Meanwhile, after removing the interbatch differences between GSE1869 and GSE5406, the new integrated gene expression profiles were used as validation sets to verify the expression levels of the key genes.

### Identification of DEGs

The DEGs between the ICM and control samples were identified using the “limma” package of R software [55]. The threshold values were *p* < 0.05 and |log fold change (FC)| > 1. The “heatmap” and “ggplot2” R packages were used to draw heatmaps and volcano plots.

### Functional enrichment analysis of DEGs

The “enrichplot” R package was used to conduct GO and KEGG pathway analyses [56]. Cell composition (CC), biological processes (BP), and molecular functions (MF) were included in the GO analysis. Then, DO analysis was performed using the “clusterProfiler” R package [56]. A *p* value < 0.05 was considered statistically significant.

### Screening key DEGs

The “randomForest” R package was used to construct a random forest classification model to screen the key DEGs associated with ICM [57]. The Gini index was used as an importance measure [58], and the best number of trees in the random forest algorithm was set to 450. Since the expression of the ninth gene was not detected in the subsequent validation set, the ninth gene could not be further validated in the validation set. So that, the genes with importance values greater than 2 [57, 59] and ranking in the top eight were considered the key DEGs of ICM for subsequent model construction. The key genes screened in the training sets were reclassified into unsupervised hierarchical clusters using the “pheatmap” software package, and a heatmap was plotted.

### Construction and verification of the ICM diagnostic signature and nomogram

A nomogram [60] was used to demonstrate the diagnostic efficacy of a diagnostic model based on eight key genes to distinguish ICM from healthy subjects. We visualized the ICM prediction model using the nomogram method and scored the expression values of the top 8 key genes. The scores of the 8 key genes were then summed to obtain the total score. The value of the risk of ICM can be determined by drawing a vertical line at the total score obtained. To quantify the nomogram prediction function, a calibration curve was plotted to evaluate the consistency between the predictions of the nomograms and the actual observations and to describe the calibration of the model according to the consistency between the predicted risk and the actual outcome in the training and testing sets. Decision curve analysis (DCA) was conducted to evaluate the net clinical benefit of the model for predicting ICM in the training and testing sets, and the model had diagnostic value if the drawn DCA curve was higher than the horizontal and dotted lines. Subsequently, a clinical impact curve was drawn to assess the clinical practicability and applicability net benefits of the nomogram model in the training and testing sets. The “ROC” package was used to draw receiver operator characteristic (ROC) curves of the nomogram in the training and validation sets to evaluate the diagnostic efficacy of the nomogram.

### Correlation between key genes and immune cells

The immune infiltration pattern in the ICM was determined using the “CIBERSORT” software package [18]. The “ggplot2” package in R software was used to draw the boxplot, heatmaps, and histograms after obtaining the immune cell expression matrix. The histogram suggests the percentage of 22 immune cells infiltrated in ICM samples, while the difference in immune cell infiltration between the ICM and control samples was displayed through boxplot and heatmap diagrams. The “corrplot” software package was used to calculate the correlation coefficient between the 8 key genes and each immune cell and visualize the results by the relevant heatmap.

### Study population

A total of 60 participants were recruited from Hunan Provincial People’s Hospital, including 30 ICM patients and 30 healthy participants. The diagnostic criteria for ICM are as follows: a history of myocardial infarction, coronary revascularization (coronary artery bypass grafting or percutaneous coronary intervention), ≥ 75% stenosis of the left main or proximal left anterior descending coronary artery, or ≥ 75% stenosis of ≥ 2 epicardial vessels [61]. All enrolled ICM patients had a left ventricular ejection fraction of ≤ 40% and a history of symptomatic heart failure (New York Heart Association [NYHA] functional class II or greater) [62]. Patients with a history of haematological disease, idiopathic cardiomyopathy, autoimmune disease, neoplasia, and severe renal or liver insufficiency were excluded. All participants provided written informed consent before the beginning of the study.

### Validation of the top 8 key DEGs in the testing set and the collected clinical samples

First, boxplots were drawn to compare the relative expression levels of the top 8 key genes between ICM and the control samples in the testing set (GSE1869 combined with GSE5406). Then, RT-qPCR analysis was used to compare the relative expression levels of the top 8 key genes between ICM and the control samples. Total RNA was obtained from peripheral blood samples of the patients using a RNeasy™ Mini Kit (QIAGEN, Frankfurt, Germany). The total RNA was reverse transcribed into cDNA using the PrimeScript RT reagent kit (Takara Bio, Japan). RT-qPCR was performed with a Taq PCR Master Mix Kit (Takara, Otsu, Japan) using an ABI 7500 instrument (Applied Biosystems, USA).

### Statistical analysis

R software (version 4.6.0) was used to perform the bioinformatics analyses. ROC curve analyses were performed using GraphPad Prism software (version 9.0.0). A p < 0.05 was considered statistically significant.

## Supplementary Material

Supplementary Figures

Supplementary Table 1

Supplementary Table 2

Supplementary Table 3

Supplementary Table 4

Supplementary Table 5

Supplementary Table 6
