# Supplementary material for: Identification MNS1, FRZB, OGN, LUM, SERP1NA3 and FCN3 as the potential immune-related key genes involved in ischaemic cardiomyopathy by random forest and nomogram
Source: Aging (Albany NY). 2023 Feb 27;15(5):1475–95. doi: 10.18632/aging.204547 (PMC10042686; doi:10.18632/aging.204547)
Supplement: Supplementary Figures [file aging-15-204547-s001.pdf]

SUPPLEMENTARY FIGURES

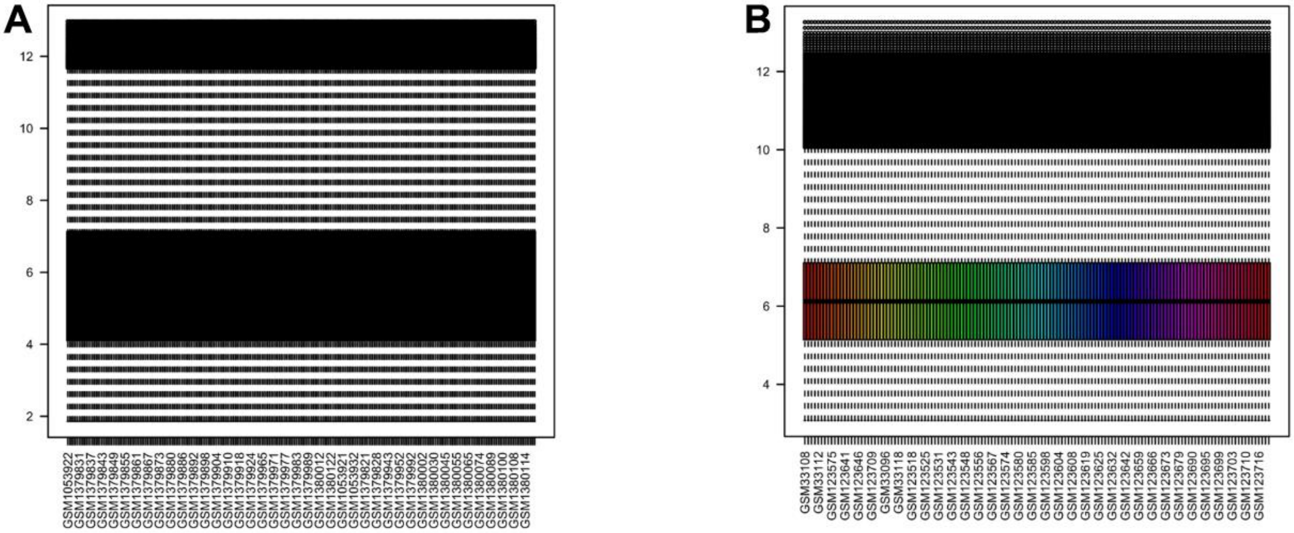

Supplementary Figure 1. Normalized for all the samples. (**A**) shows GSE42955 combined with GSE57338 and (**B**) shows GSE1869 combined with GSE5406.

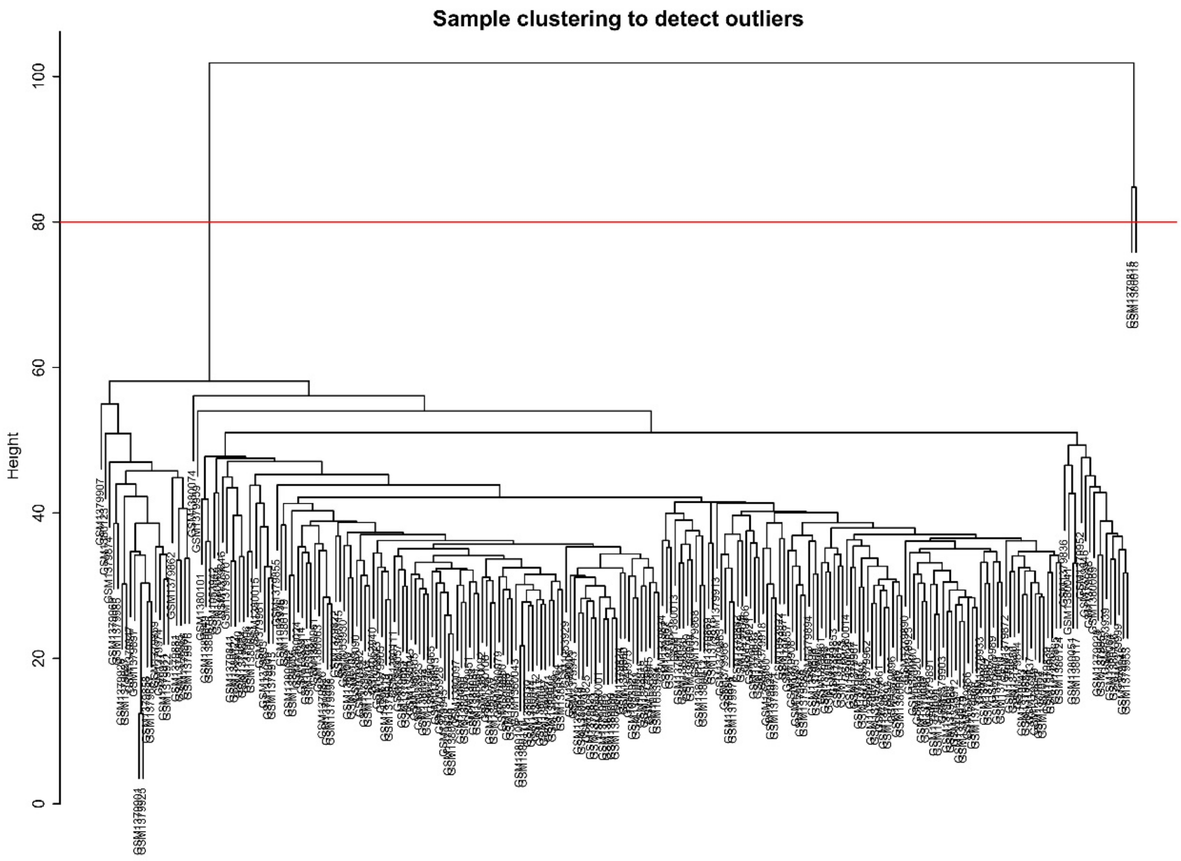

Supplementary Figure 2. Clustering dendrogram of samples. Cut height = 80 was used to divide 248 samples in the training sets (GSE42955 combined with GSE57338) into two different cluster types. Cluster 0 contains the following two samples (GSM1379815 and GSE1380018), cluster 1 contains the following 246 samples (GSM1053922, GSM1053929, GSM1053939, GSM1053940, GSM1053942, GSM1379830, GSM1379831, GSM1379832, GSM1379833, GSM1379834, GSM1379835, GSM1379836, GSM1379837, GSM1379838, GSM1379839, GSM1379840, GSM1379841, GSM1379842, GSM1379843, GSM1379844, GSM1379845, GSM1379846, GSM1379847, GSM1379848, GSM1379849, GSM1379850, GSM1379851, GSM1379852, GSM1379853, GSM1379854, GSM1379855, GSM1379856, GSM1379857, GSM1379858, GSM1379859, GSM1379860, GSM1379861, GSM1379862, GSM1379863, GSM1379864, GSM1379865, GSM1379866, GSM1379867, GSM1379868, GSM1379869, GSM1379870, GSM1379871, GSM1379872, GSM1379873, GSM1379874, GSM1379875, GSM1379876, GSM1379877, GM1379878, GM1379880, GSM1379881, GSM1379882, GSM1379883, GSM1379884, GSM1379885, GSM1379886, GSM1379887, GSM1379888, GSM1379889, GSM1379890, GSM1379891, GSM1379892, GSM1379893, GSM1379894, GSM1379895, GSM1379896, GSM1379897, GSM1379898, GSM1379899, GSM1379900, GSM1379901, GSM1379902, GSM1379903, GSM1379904, GSM1379905, GSM1379906, GSM1379907, GSM1379908, GSM1379909, GSM1379910, GSM1379911, GSM1379912, GSM1379913, GSM1379914, GSM1379915, GSM1379918, GSM1379919, GSM1379920, GSM1379921, GSM1379922, GSM1379923, GSM1379924, GSM1379925, GSM1379926, GSM1379927, GSM1379961, GSM1379962, GSM1379965, GSM1379966, GSM1379967, GSM1379968, GSM1379969, GSM1379970, GSM1379971, GSM1379972, GSM1379973, GSM1379974, GSM1379975, GSM1379976, GSM1379977, GSM1379978, GSM1379979, GSM1379980, GSM1379981, GSM1379982, GSM1379983, GSM1379984, GSM1379985, GSM1379986, GSM1379987, GSM1379988, GSM1379989, GSM1379990, GSM1379991, GSM1380008, GSM1380010, GSM1380011, GSM1380012, GSM1380013, GSM1380014, GSM1380015, GSM1380021, GSM1380027, GSM1380122, GSM1380123, GSM1380124, GSM1053914, GSM1053916, GSM1053920, GSM1053921, GSM1053923, GSM1053927, GSM1053928, GSM1053930, GSM1053931, GSM1053932, GSM1053934, GSM1053936, GSM1379813, GSM1379814, GSM1379819, GSM1379821, GSM1379822, GSM1379823, GSM1379824, GSM1379825, GSM1379826, GSM1379828, GSM1379829, GSM1379928, GSM1379930, GSM1379933, GSM1379938, GSM1379943, GSM1379945, GSM1379946, GSM1379948, GSM1379949, GSM1379951, GSM1379952, GSM1379953, GSM1379958, GSM1379959, GSM1379960, GSM1379964, GSM1379992, GSM1379997, GSM1379998, GSM1379999, GSM1380000, GSM1380001, GSM1380002, GSM1380004, GSM1380005, GSM1380007, GSM1380019, GSM1380024, GSM1380030, GSM1380040, GSM1380041, GSM1380042, GSM1380043, GSM1380044, GSM1380045, GSM1380047, GSM1380049, GSM1380051, GSM1380052, GSM1380054, GSM1380055, GSM1380057, GSM1380060, GSM1380061, GSM1380063, GSM1380064, GSM1380065, GSM1380066, GSM1380067, GSM1380069, GSM1380070, GSM1380071, GSM1380074, GSM1380076, GSM1380079, GSM1380081, GSM1380086, GSM1380087, GSM1380089, GSM1380090, GSM1380091, GSM1380092, GSM1380093, GSM1380097, GSM1380100, GSM1380101, GSM1380104, GSM1380105, GSM1380106, GSM1380107, GSM1380108, GSM1380109, GSM1380110, GSM1380111, GSM1380112, GSM1380113, GSM1380114, GSM1380115, GSM1380116, GSM1380117, GSM1380119 and GSM1380120).

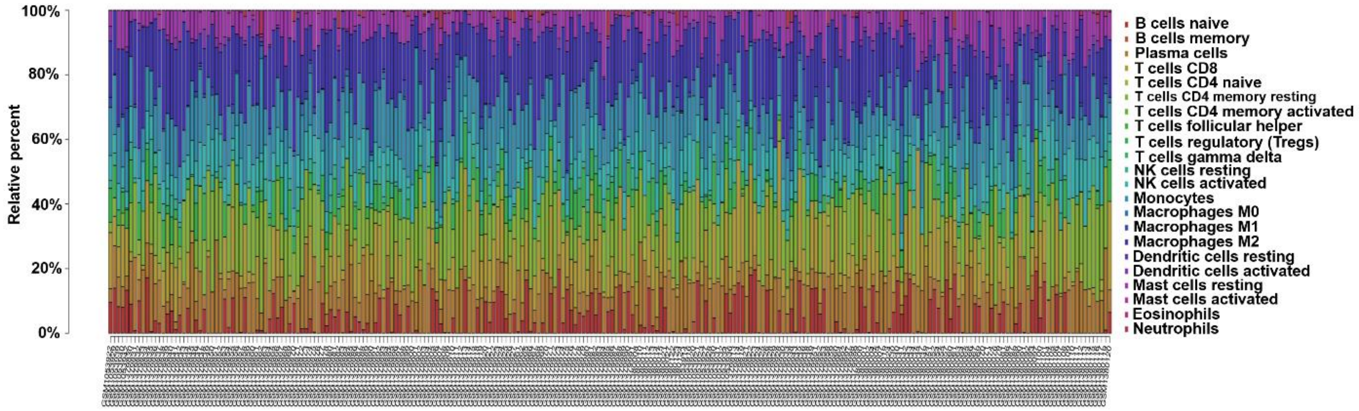

Supplementary Figure 3. Infiltration pattern of immune cell subtypes in validation set. The bar plot visualizing the relative percent of 22 immune cell in each sample.

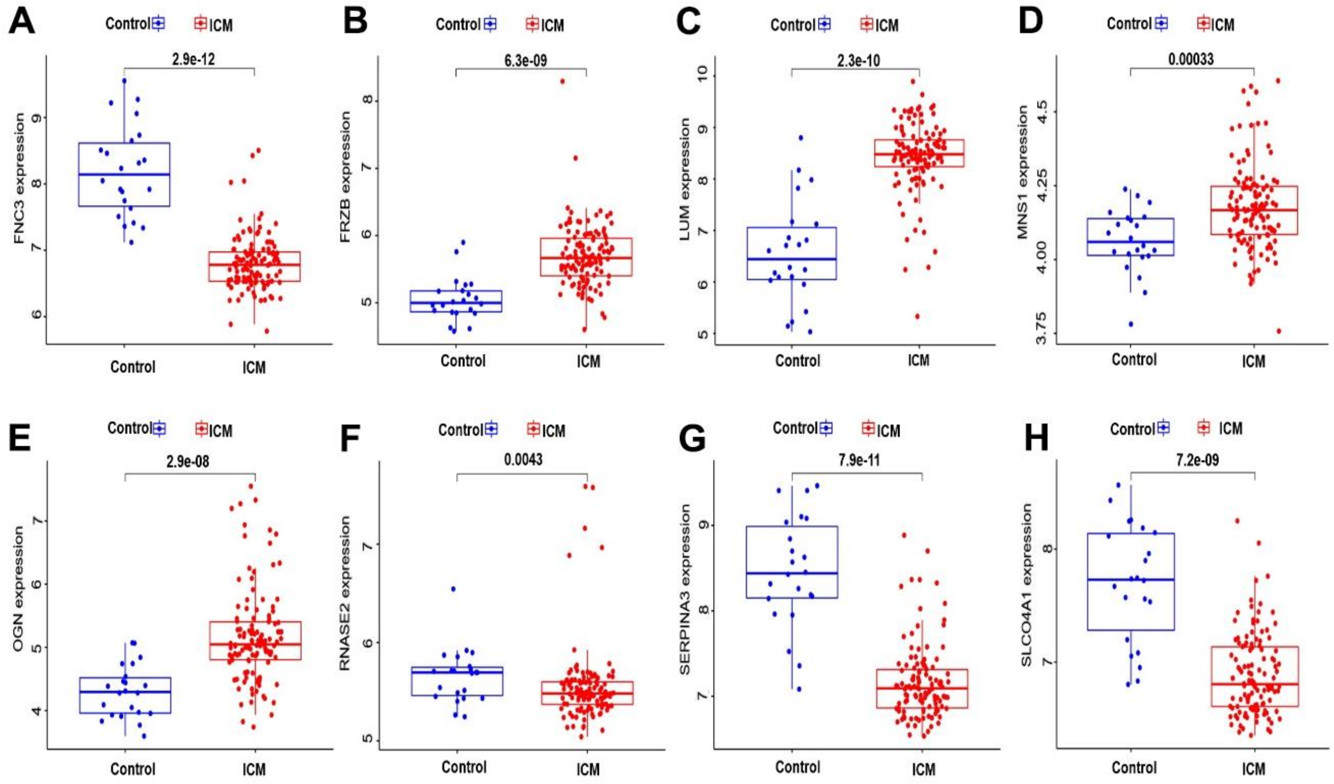

Supplementary Figure 4. External validation of the key genes in testing set. The expression levels of FCN3 (**A**), FRZB (**B**), LUM (**C**), MNS1 (**D**), OGN (**E**), RNASE2 (**F**), SERPINA3 (**G**), SLCO4A1 (**H**) in testing set.
