# Supplementary material for: Identification MNS1, FRZB, OGN, LUM, SERP1NA3 and FCN3 as the potential immune-related key genes involved in ischaemic cardiomyopathy by random forest and nomogram
Source: Aging (Albany NY). 2023 Feb 27;15(5):1475–95. doi: 10.18632/aging.204547 (PMC10042686; doi:10.18632/aging.204547)
Supplement: Supplementary Table 3 [file aging-15-204547-s004.pdf]

Supplementary Table 3. The importance of 39 DEGs.

Gene	Importance
FCN3	19.31250056
SERPINA3	19.25873157
MNS1	7.778561915
OGN	7.202295921
SLCO4A1	5.872656897
RNASE2	5.399599675
FRZB	5.037672655
LUM	4.95926197
SMOC2	4.958591477
SFRP4	4.116572428
VSIG4	4.024444085
CD163	3.813078851
FCER1G	2.852484032
ADAMTS9	2.749976824
ASPN	2.486912883
PHLDA1	1.847027445
MYOT	1.836173723
ANKRD2	1.743026675
LYVE1	1.677558479
IL1RL1	1.466302701
HBB	1.209413475
MYH6	1.156130419
PLA2G2A	1.012830974
C6	0.805501497
CYP4B1	0.76590613
MGST1	0.689610082
ADAMTS4	0.636695692
NPPA	0.627402182
MXRA5	0.616162724
UTY	0.508651928
DDX3Y	0.49215963
USP9Y	0.462653587
SERPINE1	0.436366429
HMGCS2	0.422313019
EIF1AY	0.38407382
MIR208A	0.371855071
AOX1	0.333867857
IFI44L	0.305582353
DSC1	0.269703898
